# Posttranscriptional (Re)programming of Cell Fate: Examples in Stem Cells, Progenitor, and Differentiated Cells

**DOI:** 10.3389/fimmu.2018.00715

**Published:** 2018-04-09

**Authors:** Chrysi Kanellopoulou, Stefan A. Muljo

**Affiliations:** Laboratory of Immunology, National Institute of Allergy and Infectious Diseases, National Institutes of Health, Bethesda, MD, United States

**Keywords:** posttranscriptional regulation, RNA-binding protein, microRNA, embryonic stem cell, Th17, fetal hematopoiesis, gene regulatory network, hematopoietic stem and progenitor cells

## Abstract

How a single genome can give rise to many different transcriptomes and thus all the different cell lineages in the human body is a fundamental question in biology. While signaling pathways, transcription factors, and chromatin architecture, to name a few determinants, have been established to play critical roles, recently, there is a growing appreciation of the roles of non-coding RNAs and RNA-binding proteins in controlling cell fates posttranscriptionally. Thus, it is vital that these emerging players are also integrated into models of gene regulatory networks that underlie programs of cellular differentiation. Sometimes, we can leverage knowledge about such posttranscriptional circuits to reprogram patterns of gene expression in meaningful ways. Here, we review three examples from our work.

## Introduction

The sequencing of the first human genome (1), in principle, provided us with a complete parts list and blueprint for building a human being. However, our work is far from done, and a quote from Richard Feynman applies here: “What I cannot create, I do not understand.” This is a daunting challenge for biologists, since we know little about how all these parts fit and work together to make a functional human cell, the basic unit of life. Furthermore, it has been estimated that an average adult human being is composed of 30–37 trillion cells (2, 3). How a single-cell embryo can give rise to all these cells and ultimately a whole organism is still poorly understood.

**Figure 1:**
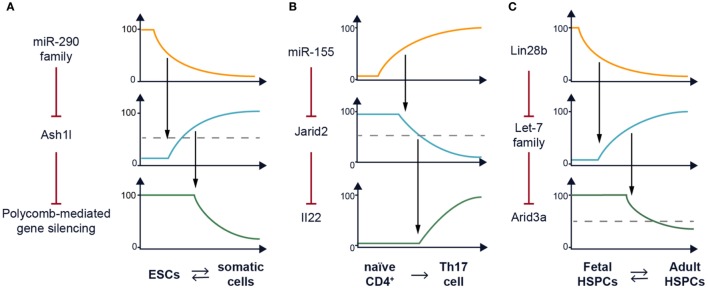
Recurrent network motif in posttranscriptional (re)programming. **(A)** The miR-290–Ash1l–polycomb repressive complex 2 (PRC2) axis plays a role in setting the chromatin landscape of embryonic stem cells (ESCs) to support the pluripotency gene expression program. A model of how the expression of the miR-290 family and Ash1l varies to impact activity of PRC2 is depicted along a time course as ESCs undergo differentiation. This process is reversible (7–9). **(B)** The miR-155–Jarid2 axis can also remodel the chromatin landscape by regulating PRC2 recruitment to support the Th17 gene expression program including transcription of the *Il22* cytokine gene among many others. A model of how the expression of miR-155 and Jarid2 varies to impact *Il22* transcription is depicted along a time course as naïve CD4^+^ T cells undergo Th17 differentiation. This process has not been shown to be reversible. **(C)** The Lin28b–let-7 axis mediates the fetal–adult hematopoietic switch. One downstream target of this pathway in B cell lineage progenitors is *Arid3a* messenger RNA which encodes a transcription factor (10). A model of how the expression of Lin28b and the let-7 family varies to impact *Arid3a* posttranscriptionally is depicted for hematopoietic stem and progenitor cells (HSPCs) during ontogeny. This process is reversible (11).

The answer must be contained in the genome if we could fully decode it. First, the central dogma of molecular biology posits that DNA (the genome) is transcribed into RNA (the transcriptome) and then translated into protein (the proteome) (4). Thus, RNA has been considered mainly as a “messenger” to transmit information encoded in the genome to produce the proteome. However, even if we understood the function of all the proteins encoded by our DNA that would only account for ~1% of the information content of the genome (1). That leaves the bulk of the genome, presumably harboring the blueprint for life, that we are only beginning to understand. For example, a part of the blueprint that is best understood contains instructions for the transcriptional machinery to either switch genes on or off. Indeed, gene regulation at the DNA level within the cell’s nucleus is an active and exciting field of research. However, it has come to light that RNA does not only serve as a template to encode protein, also known as messenger RNA (mRNA). It turns out that most of the genome (~75%) is transcribed, in other words, able to generate complementary RNA (5), but these transcripts are not always translated giving birth to the field of “non-coding RNAs.”^1^ As such, the number of annotated non-coding RNAs rivals the number of protein-coding transcripts (6), and we will need to determine what functions these factors of emerging importance play. To draft an outline of a working roadmap for putting all these parts together, system biologists have begun mapping various types of networks to catalog as comprehensively as possible how diverse biomolecules interact with each other. We are doing our small part to begin integrating the roles of regulatory non-coding RNAs and associated RNA-binding proteins in this larger framework. We have noticed a recurring theme from our work (Figure 1).

## Inducing Pluripotency by Posttranscriptional Reprogramming

If we knew the genetic programs underlying cell fate specification, it would be possible to instruct cells to perform desired biological functions at will. For example, Takahashi and Yamanaka employed four transcription factors to instruct mature somatic cells to de-differentiate back to an embryonic-like pluripotent stem cell state (7). Interestingly, two independent groups found that they could accomplish this feat in cellular reprogramming using a class of small (19–23 nucleotides long) non-coding RNAs called microRNAs (miRNAs) (8, 9). This represents one example of posttranscriptional reprogramming; however, the mechanisms of action are not well understood.

**Figure 2:**
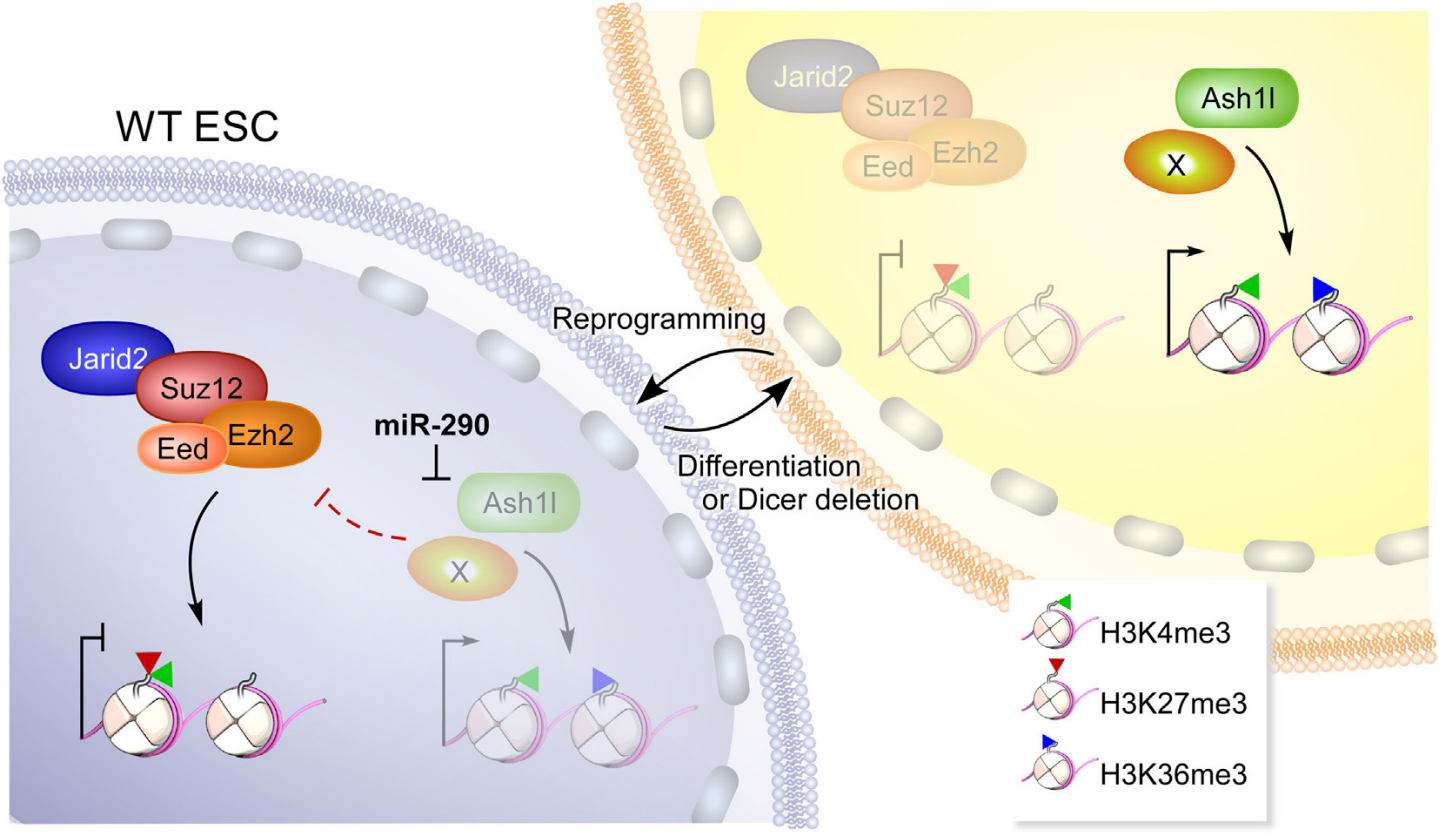
A model depicting how miR-290 reprograms the epigenome of embryonic stem cells (ESCs). In wild-type (WT) ESCs, high levels of miR-290 represses Ash1l and perhaps additional targets (depicted by “X”) that can otherwise antagonize polycomb repressive complex 2 (PRC2) (containing Ezh2, Eed, Suz12, and Jarid2). PRC2 activity results in deposition of H3K27me3 marks on chromatin including bivalent domains that harbor H3K4me3 (20), a mark on active or poised promoters. Upon Dicer deletion or differentiation, miR-290 levels are reduced and can no longer repress Ash1l and additional targets. Unfettered, Ash1l activity results in increased H3K36me3 marks and antagonizes PRC2 (17). This figure is reproduced from Kanellopoulou et al. (14).

We have previously reported that ablation of Dicer, the RNAse III-containing enzyme required for miRNA processing impairs mouse embryonic stem cell (ESC) differentiation and self-renewal (12). Furthermore, Dicer is required for the generation of induced pluripotent stem cells (13). A reasonable candidate for mediating these activities is the miR-290 family (14), a miRNA cluster that is highly expressed in mouse ESCs and is downregulated during differentiation. Interestingly, the miR-290 locus has one of the top ranked super enhancers in ESCs (seventh out of 231), higher than the pluripotency genes encoding Oct-4 and Nanog (15). miRNAs target complementary mRNAs by base pairing, *via* their so called seed sequence, a six to eight nucleotide motif at their 5′ end (16). Members of miR-290 share the same seed sequence as the miR-302 family used in the two studies mentioned earlier and therefore are predicted to target the same mRNAs. We determined that expression of the Trithorax group protein Ash1l is posttranscriptionally repressed by these ESC-specific miRNAs (Figure 1A) (14). Ash1l is a methyltransferase which promotes tri-methylation of histone H3 at lysine 36 (H3K36me3), an epigenetic mark associated with ongoing gene transcription. One function of Ash1l is to antagonize Polycomb-mediated gene silencing (17). The polycomb repressive complex 2 (PRC2) catalyzes tri-methylation of H3K27, a histone mark associated with silencing. PRC2 has been shown to be essential for pluripotency maintenance and induction (18, 19). Indeed, we found that in the absence of miRNAs, the *Homeobox* (*Hox*) gene clusters, which are canonical targets of PRC2, have reduced H3K27me3 marks and PRC2 occupancy and are de-repressed. This defect in epigenetic silencing could be rescued by transfection of a single representative member of the miR-290 family (14, 20). Furthermore, this defect can also be rescued by Ash1l knockdown (14). A similar study, showing defective polycomb recruitment in the absence of miR-290, was independently performed by Graham et al. (20) further confirming the importance of this family of miRNAs in ESC pluripotency. In summary, a single miRNA family can reprogram the epigenetic landscape of a cell. By affecting the balance between H3K36me3 and H3K27me3, miR-290 can promote the pluripotent program of gene expression (Figure 2).

## Reprogramming Chromatin in Th17 Cells

This general principle can be used in any cellular differentiation system. Indeed, we found a second similar example; although, it was not the original motivation of our work to demonstrate the generality of our idea. We screened for potentially interesting miRNAs in mouse T helper cell differentiation and found that miR-155 is highly expressed in Th17 cells compared with other subsets. Expression of miR-155 was induced upon T cell activation and was highly dependent on addition of IL-6 and IL-1β (21). Furthermore, we found that these two cytokines synergistically activated miR-155 expression in Th17 cells in a Stat3-dependent manner (21, 22), and later realized that the *Mir155* locus harbored a super enhancer (23). Our investigations further revealed that this miRNA also plays a role in programming the epigenetic landscape in Th17 cells (24). In the absence of miR-155, there is increased recruitment of PRC2 to thousands of locations in the genome, and enhanced tri-methylation of H3K27 at those sites. While Th17 cell differentiation still occurs in the absence of miR-155, we found significant defects in cytokine gene expression, a vital function of Th17 cells. In miR-155 knockout mice, we found CD4^+^RORγt^+^ Th17 cells *in vivo*, but they displayed a significant cell-intrinsic defect in IL-17 and IL-22 expression (24).

We determined that the root of the problem is de-repression of Jarid2, a target of miR-155 in Th17 cells (Figure 1B), and a key component of PRC2. It was recently found that Jarid2 is essential for recruitment of PRC2 to chromatin (25–29). Indeed, the defect in cytokine gene expression by Th17 cells can be rescued partially by deleting just one allele of Jarid2, thus reducing its expression by 50%. The partial rescue we observed with the compound deletion of miR-155 and Jarid2 highlights the fact that miRNAs target multiple transcripts and often it is hard to identify a single target that can restore the dysregulation of an miRNA deficiency. In that same experiment, we also observed genetic epistasis between miR-155 and Jarid2 with regards to homeostasis of Foxp3^+^ T regulatory cells indicating that this regulatory circuit is used again in a different context. Thus, the concentration of Jarid2 can be used to modulate the global activity of polycomb-mediated gene silencing, and we have uncovered a situation in which miR-155 has co-opted this function as a rheostat.

## Lin28b-Mediated Reprogramming in Hematopoiesis

In a third project, we screened for miRNAs that distinguished progenitor B (pro-B) cells isolated from fetal liver versus adult bone marrow. The let-7 family of miRNAs is highly expressed in pro-B cells from adult bone marrow but not fetal liver (11). Since the different let-7 members are encoded by seven disparate genetic loci, it seems unlikely that this differential expression is regulated transcriptionally. Rather we postulated that there could be posttranscriptional regulation of the whole family. An RNA-binding protein, Lin28, had already been discovered to inhibit maturation of let-7 miRNAs (30), was a likely candidate (Figure 1C). In support of our hypothesis, we found that Lin28b, one of two paralogs, is highly expressed in fetal hematopoietic stem and progenitor cells (HSPCs) but not in their adult counterparts. Furthermore, enforced expression of Lin28 in adult HSPCs reprogrammed lymphocyte development to mimic fetal ontogeny. As evidence that we have uncovered a general molecular mechanism for fetal–adult hematopoietic switching, ectopic expression of LIN28B in adult erythroblasts is also sufficient to turn on fetal hemoglobin expression (31). This provides a novel avenue for the treatment of beta-thalassemia and sickle cell disease that may avoid the cytotoxic effects of hydroxyurea, currently the only clinically approved treatment for beta-globinopathies. Furthermore, we hope to inspire a new and better strategy to regenerate the hematopoietic and immune system. Specifically, Lin28b-reprogrammed HSPCs may be useful for transplantation in neonates or *in utero* if adult hematopoietic stem cells could be rejuvenated to become fetal again.

On a personal note, Bill Paul would frequently ask whether we had looked at embryonic-derived macrophages and whether their specification might also depend on Lin28b. Sadly, we failed to provide Bill with an answer before he passed away, but we are working hard to determine whether Lin28b also (re)programs myeloid lineages, in addition to lymphoid and erythroid differentiation in memory of his inquisitiveness.

## Conclusion

Overall, these studies support the idea that studying posttranscriptional regulatory networks will not only reveal interesting molecular mechanisms for controlling gene expression programs but can also provide novel therapeutic targets for reprogramming cell fates.

## Author Contributions

All authors listed have made a substantial, direct, and intellectual contribution to the work and approved it for publication.

## Conflict of Interest Statement

The authors declare that the research was conducted in the absence of any commercial or financial relationships that could be construed as a potential conflict of interest.

## Footnotes

^1^This popular term in an unfortunate misnomer because it suggests that these RNAs do not harbor genetic code. It would have been more accurate to call them untranslated RNAs rather than non-coding RNAs.
